# Myocardial infarct size quantification in mice by SPECT using a novel algorithm independent of a normal perfusion database

**DOI:** 10.1186/2191-219X-2-64

**Published:** 2012-12-28

**Authors:** Véronique Roelants, Marie Delgaudine, Stephan Walrand, Renaud Lhommel, Yves Beguin, François Jamar, Jean-Louis Vanoverschelde

**Affiliations:** 1 Institut de Recherche Expérimentale et Clinique, Pôle d’Imagerie Moléculaire, Radiothérapie et Oncologie and Pôle de Recherche Cardiovasculaire, Université Catholique de Louvain, Brussels, 1200, Belgium; 2 Division of Hematology, University of Liège, Liege, 4000, Belgium; 3 Institut de Recherche Expérimentale et Clinique, Pôle d’Imagerie Moléculaire, Radiothérapie et Oncologie, Université Catholique de Louvain, Brussels, 1200, Belgium; 4 Institut de Recherche Expérimentale et Clinique, Pôle Cardiovasculaire, Université Catholique de Louvain, Brussels, 1200, Belgium; 5 Cliniques Universitaires Saint-Luc, Department of Nuclear Medicine, Avenue Hippocrate 10, Brussels, 1200, Belgium

**Keywords:** Infarct size, Quantification, Mouse, SPECT

## Abstract

**Background:**

There is a growing interest in developing non-invasive imaging techniques permitting infarct size (IS) measurements in mice. The aim of this study was to validate the high-resolution rodent Linoview single photon emission computed tomography (SPECT) system for non-invasive measurements of IS in mice by using a novel algorithm independent of a normal database, in comparison with histology.

**Methods:**

Eleven mice underwent a left coronary artery ligature. Seven days later, animals were imaged on the SPECT 2h30 after injection of 173 ± 27 MBq of Tc-99m-sestamibi. Mice were subsequently killed, and their hearts were excised for IS determination with triphenyltetrazolium chloride (TTC) staining. SPECT images were reconstructed using the expectation maximization maximum likelihood algorithm, and the IS was calculated using a novel algorithm applied on the 20-segment polar map provided by the commercially available QPS software (Cedars-Sinai Medical Center, CA, USA). This original method is attractive by the fact that it does not require the implementation of a normal perfusion database.

**Results:**

Reconstructed images allowed a clear delineation of the left ventricles borders in all mice. No significant difference was found between mean IS determined by SPECT and by TTC staining [37.9 ± 17.5% vs 35.6 ± 17.2%, respectively (*P* = 0.10)]. Linear regression analysis showed an excellent correlation between IS measured on the SPECT images and IS obtained with TTC staining (*y* = 0.95x + 0.03 (*r* = 0.97; *P* < 0.0001)), without bias, as demonstrated by the Bland-Altman plot.

**Conclusion:**

Our results demonstrate the accuracy of the method for the measurement of myocardial IS in mice with the Linoview SPECT system.

## Background

Animal models of myocardial infarction are commonly used to investigate pathophysiological mechanisms and to evaluate strategies aimed at reducing infarct size [1]. Genetically modified mice are increasingly used for this purpose [2]. Until recently, quantification of myocardial infarct size in mice was only possible by use of post-mortem histological analysis. Although histological assessment of infarct size is well validated and considered to be the golden standard for infarct size quantification, it requires euthanizing the animal and thus precludes intra-animal infarct size follow-up. It also makes it difficult to conduct longitudinal studies, as these require euthanizing numerous animals at multiple time points to obtain the desired temporal information [3]. A non-invasive, non-destructive method for estimating infarct size would thus be most desirable.

In patients with coronary artery disease, accurate quantification of infarct size can be obtained using single photon computed emission tomography (SPECT) and Tc-99m-labelled perfusion tracers [4]. This method has been validated in large animal models of myocardial infarction and can be used to serially measure infarct size in the same individuals. Although theoretically feasible, application of this methodology to mice imaging is challenging, owing to the very small size of the mouse heart. The main challenge when imaging the mouse hearts is to achieve spatial resolution and noise levels that permit resolving the millimetre-thick murine myocardial wall. Earlier investigators have nonetheless succeeded in imaging rodent heart with small animals SPECT or positron-emission tomography (PET) cameras [5-11] and reported reasonably good correlations between infarct size measured by these modalities and that obtained with histology [7-11].

The Linoview SPECT system is a novel whole-body high-resolution SPECT camera that was recently developed to image rodents [12]. Compared with other dedicated small-animal SPECT imaging systems, the system uses focusing collimators moved along linear orbits providing linogram acquisitions. In this setup, the tomographic field of view is determined by the orbit length independently of the collimator acceptance angle. As a consequence, the collimator aperture can be set closer to the animal, without any truncation issues, which results in increased efficiency and spatial resolution (0.6 mm). In this study, we used this system to investigate the feasibility of assessing myocardial perfusion distribution and infarct size in mice with prior myocardial infarction. Total perfusion deficit (TPD) index developed by Slomka et al. [13] that combines defect severity and extent in one parameter was compared with triphenyltetrazolium chloride (TTC) histological data. TPD is based on the comparison of the polar map voxels to normal limits derived from a normal perfusion database. In addition, a novel algorithm that does not require use of a normal perfusion database was developed in order to provide an absolute infarcted fraction (AIF).

## Methods

### Mouse model of myocardial infarction

Animal experiments were performed in accordance with institutional guidelines and approved by the Animal Care Ethical Committee of the University of Liège. Myocardial infarction was induced in 2-month old female C57Bl/6 mice (18.5 ± 0.9 g). Anaesthesia was induced and maintained using ketamine (100 mg/kg) and xylazine (5 mg/kg). Mice were orally intubated with a 22-gauge vinyl catheter, and positive pressure ventilation (0.3 ml/min, 160 breaths/min) was maintained with a rodent ventilator (Harvard Apparatus, Les Ulis Cedex, France). The heart was exposed through a 1-cm left lateral thoracotomy. The left coronary artery (LCA) was ligatured by passing an 8-0 polypropylene silk suture around the artery. The chest wall, muscle layers and skin were then closed with interrupted 6-0 polypropylene silk sutures. Intubation was discontinued once spontaneous breathing resumed, and the mouse was allowed to recover on a heated platform.

### SPECT acquisition

A total of 11 infarcted animals and seven healthy controls were scanned. SPECT acquisitions were performed using the Linoview SPECT system (Linoview System, Amsterdam, The Netherlands) [12]. The slit width was set to 0.6 mm. Acquisitions were performed 7 days after the LCA ligature. Animals received an intravenous injection of 173 ± 27 MBq of ^99m^Tc-sestamibi through the tail vein. Two hours later, 0.1 μg/kg of cholecystokinin was injected intraperitoneally to induce emptying of the gallbladder. The animals were scanned 30 min later, under anaesthesia (50 mg/kg of ketamine and 2.5mg/kg of xylazine) for 30 min.

### SPECT data reconstruction

Data were reconstructed using the expectation maximization maximum likelihood algorithm without attenuation or resolution correction. The images were reoriented into conventional short-axis, vertical long-axis and horizontal long-axis views.

### Polar map derivation

As previously reported by Constantinesco et al. [6], an appropriate scaling factor was applied on the reconstructed images in order to approach the human reference heart volume, and a 20-segment polar map was automatically derived with the QPS software (Cedar-Sinai Medical Center, Los Angeles, USA) with constraining limits being manually adjusted [13-15]. Relative segment level was defined as the ratio of the segment value given by the QPS software with that of the segment having the highest tracer uptake.

### Absolute infracted fraction assessment

The novel proposed method for AIF determination includes two steps: the determination of the myocardial wall volume corresponding to each segment of the polar map and the determination of the contribution to each segment to the total infracted volume.

**Figure 1:**
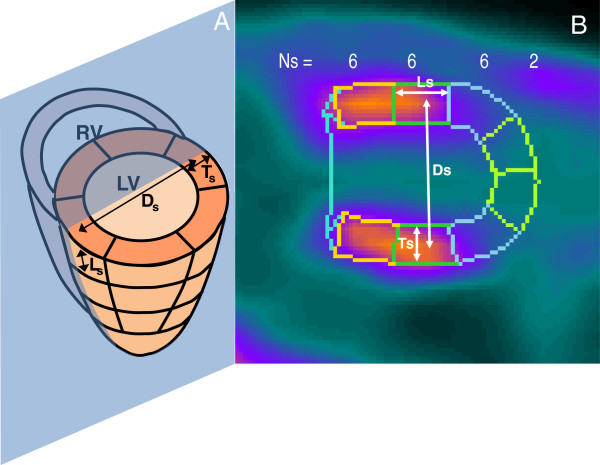
**Measurement of the segments dimensions.** Schematic (**A**) and factual (**B**) illustration of the central vertical long axis slice delimited by the QPS software on which measure were performed to determine the volume of each of the 20 segments s of the polar map. Ns is the number of segment of the polar map at each level (basal, *n* = 6 segments; middle, *n* = 6 segments; apical, *n* = 6 segment; apex, *n* = 2 segments). Ds is the mean diameter of the ring made by Ns segments at each ventricular level, Ls is the long axis length, and Ts is the transverse thickness of the segments. LV, left ventricular cavity; RV, right ventricular cavity.

Step 1. The volume of each segment was measured on the central vertical long axis slices as shown in Figure 1 where the lines are the cross section of the volume of interest drawn by QPS to derive the polar map. The volume *V*_s_ of the segments is as follows:

(1) $$V_{s}=\frac{C_{s}\times \sigma_{s}}{N_{s}}$$

(2) $$C_{s}=\pi \times D_{s}$$

(3) $$\sigma =L_{s}\times T_{s}$$

The numerator of Equation 1 is the volume of the ring made by the *N*_s_ segments at the same radials distance in the polar map. Equation 2 gives the circumference *C*_s_ of the ring assuming that the ring is circular with a mean diameter *D*_s_ measured between the centres of the two sections of the ring. σ_s_ is the area of the ring section estimated as the product of the length *L*_s_ and thickness *T*_s_. These two distances were measured starting at the middle section sides in order to take into account the non-parallelism of the sides. The number of segment *N*_s_ of the polar map at each level are illustrated in Figure 1 (basal, *n* = 6; middle, *n* = 6; apical, *n* = 6; apex, *n* = 2).

Step 2. A numerical model of the mice’s left ventricle was developed in order to determine the infarcted fraction in a segment as a function of its relative level. The ventricle was modelled by a 1-cm-length cylinder of 5.4-mm-diameter (mean diameter observed in SPECT, 5.4 ± 0.7 mm) and with a uniform active wall thickness of 1 mm. A 100% activity defect was modelled on the whole wall length and with a polar extension ranging from 0° to 240° that, in this model, corresponded to a total infarcted fraction ranging from 0% to 66%. The beating was modelled by a 0.35-mm radial oscillation of the wall associated with a longitudinal twisting of 3° [16]. The breathing was modelled by a 2-mm transverse oscillation of the whole ventricle [17]. The breathing oscillation direction was modelled perpendicularly and tangentially to the defect as well. A surrounding background with specific activity 10%, 20% and 30% of that of the non-infarcted region was modelled (19.3% ± 4.9% observed in the mice SPECT). In real SPECT, this background activity resulted from the residual vascular and extravascular pool activity and from the cross contamination of high uptaking of Tc-99m in tissues such as liver and gallbladder as well.

**Figure 2:**
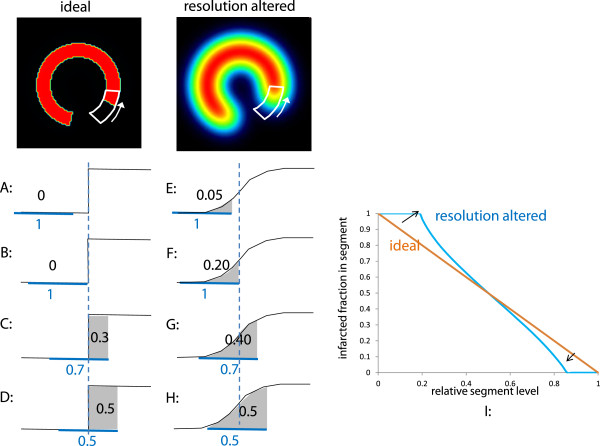
**Infarcted fraction in a segment as a function of its relative level.** A to H, evolution of the infarcted fraction in a segment (number in blue) as a function of its relative level (number in black) when the segment circumferentially travels through a defect-to-normal region edge; A to D, in case of an ideal tomography and E to H, for an altered resolution. The horizontal blue line shows the segment position. The vertical doted blue line represents the normal to defect edge position. The integration (in grey) of the activity in the segment at different polar positions gives the actual infarcted fraction in the segment as a function of its relative level (I).

After being blurred by the beating and breathing, the distribution activity was convolved with the mean tomographic 3D spatial resolution of the system which was about 1.3 mm at the heart location for the slit width set to 0.6 mm. Motion blurring and system spatial resolution spread the surrounding background activity inside the defect area. Lastly, the wall activity was summed inside a 60° arc segment circumferentially moving in order to compute its infracted fraction as a function of its relative level (Figure 2).

When a segment circumferentially moved through an ideal defect-to-normal region edge, the variation of its infarcted fraction is linear versus its relative level (A to D in Figure 2). When this step transition was smoothed by the altering resolution effects (beating, breathing and system resolution), the relative segment level already increased although the segment was still fully located in the defect area (E to F in Figure 2). The same behaviour occurred when the segment was completely inside the normal region and came close to the defect area. As a result, the curve of the infarcted fraction in segment as a function of the segment relative level underwent a pivoting (I in Figure 2).

To account for these features, a weighting factor *P*(*U*_s_) giving the infarcted fraction in a segment as a function of its relative level *U*_s_ was modelled by a s-shaped curve:

(4) $$P\left(U_{s}\right)=\frac{1}{\sqrt{1+{\left(\frac{U_{s}}{U_{c}}\right)}^{2n}}}$$

*U*_c_ is the level corresponding to the inflection point of the curve and *n* determines its slope at the inflection point. The AIF of the myocardial wall was

(5) $$\mathrm{AIF}=\frac{\sum_{s=1}^{20}P\left(U_{s}\right)V_{s}}{\sum_{s=1}^{20}V_{s}}$$

The denominator of Equation 5 is the total myocardial wall volume. *U*_c_ and *n* were fitted in order to obtain the best agreement between the infarcted percentage assessed by TTC and by SPECT.

### Histological analysis

Because 2,3,5-triphenyl tetrazolium chloride or TTC staining is coupled with mitochondrial metabolism and Tc-99m-sestamibi is sequestered in mitochondria of viable myocardium, infarct size estimated by Linoview SPECT was compared to TTC staining [18,19] which was performed as previously described [20]. Briefly, after SPECT imaging, the animals were euthanized, their chest was opened, and their heart was excised and sliced into serial 1-mm-thick short-axis slices, parallel to the atrioventricular groove. The myocardial slices were weighted and further incubated at 37°C in 1% TTC for 20 min. To avoid any issue of contrast lost or chromatographic change that can occur during digitalization, the boundaries between the infarcted and viable myocardium regions were manually drawn using a binocular magnifier on a transparent sheet directly set in contact with the heart slices. Afterwards, the transparent sheets were scanned and the area of the regions was assessed using the Image J software (Bethesda, MD, USA). With this method, the epicardial and endocardial borders of each slice as well as the contours of the infarcted area were hand-traced and expressed in percentage of the total slice area. The weight of the infarcted and non-infarcted myocardium was then calculated by multiplying their relative proportion by the weight of the corresponding myocardial slice. The infarct size was then calculated as the sum of the infarcted segments in each individual slices and expressed in percentage of total myocardial mass. SPECT and histological analysis were performed by the Université Catholique de Louvain and the Université de Liège, respectively, with prior knowledge of the analysis of the other team.

### TPD assessment

TPD [13] was computed using QGS (AutoQUANT release 7.2, Philips Medical Systems, San Jose, CA, USA). A normal perfusion database and limits were built using the seven normal mice SPECT data.

### Statistical analysis

Results are expressed as mean ± SD. Data were compared by the paired two-tailed Student’s *t* test, by linear regression and by the Bland-Altman test. *P* < 0.05 was considered statistically significant.

## Results

### Linoview SPECT image quality

**Figure 3:**
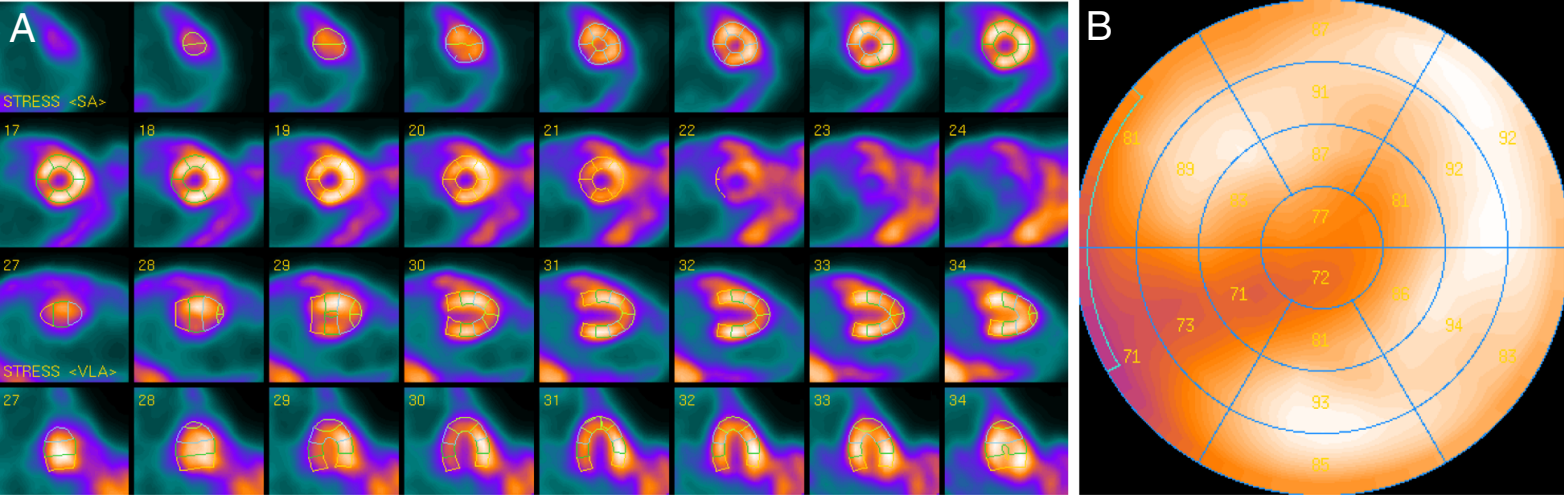
**Representative ventricular slices and the corresponding polar map of myocardial perfusion SPECT in normal mouse.** Representative short axis, horizontal long-axis, vertical long-axis slices (**A**) and the corresponding polar map (**B**) of a myocardial perfusion SPECT study of a normal mouse.

**Figure 4:**
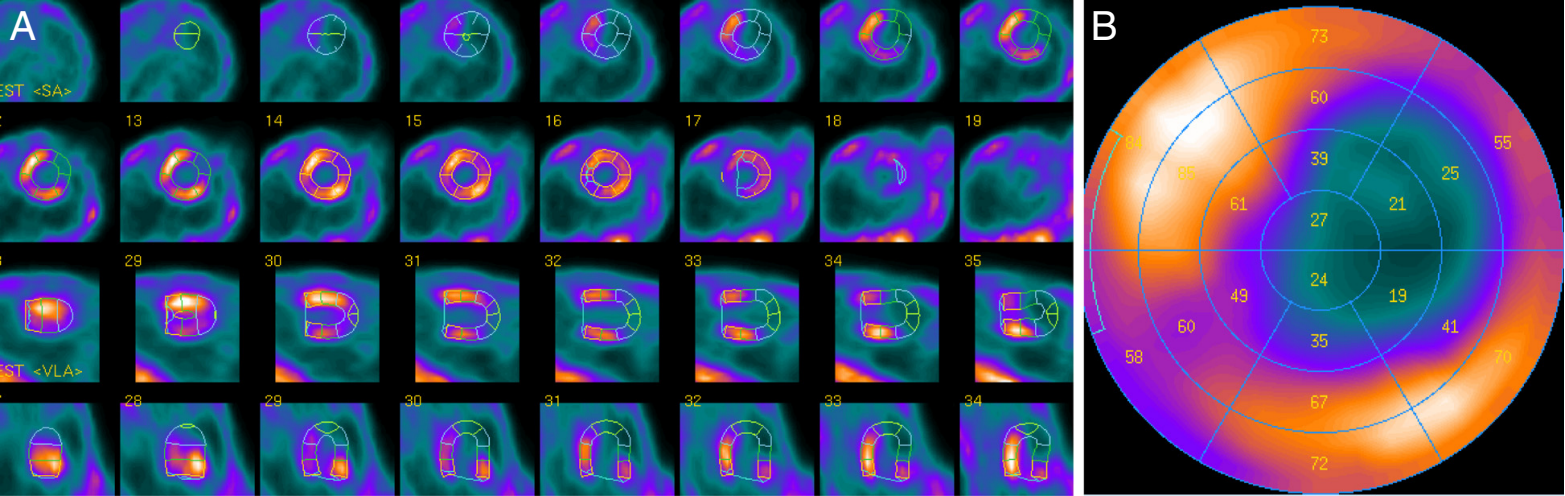
**Representative ventricular slices and corresponding polar map of myocardial perfusion SPECT in mouse with myocardial infarction.** Representative short axis, horizontal long-axis, vertical long-axis slices (**A**) and the corresponding polar map (**B**) of a myocardial perfusion SPECT study of a mouse with a myocardial infarction.

Short axis, vertical long axis, horizontal long axis slices and the corresponding polar map of a normal mouse are shown in Figure 3. The left ventricular myocardium is clearly delineated, and the thinner right ventricular myocardium is also visible on some slices. Images from a mouse with a myocardial infarction are shown in Figure 4. The perfusion defect is clearly apparent and was delineated by QPS.

### Infarcted fraction in a segment and its relative level observed in the numerical model

**Figure 5:**
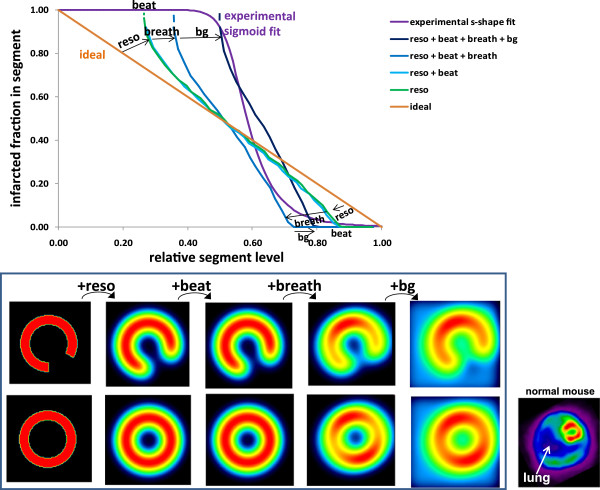
**Impact on the relative segment level of the different parameters altering the spatial resolution.** Effects of the system resolution, beating, breathing (tangential to the defect) and of the surrounding background on the infarcted fraction in a segment as a function of its relative level derived from the ventricle numerical model with a 60° polar extension defect. Note that the three first effects induced a curve clockwise pivoting, while the background induced a curve shift to the right. The corresponding transverse slices of the numerical model are shown for a pathological (top row) and for a normal modelled ventricle (bottom row). Note that the breathing induced an artifactual hypo-perfusion (image 4 at bottom row), also observed on ‘real images’ (bottom right images).

Figure 5 shows the impact of the different effects on the infarcted fraction in a segment as a function of its relative level for the 60° defect polar extension. The beating had a marginal impact. The system resolution and the breathing induced a significant clockwise pivoting of the curve, while the surrounding background induced a significant shift of the curve to the right. When the resolution is too altered, segment relative level below a certain threshold cannot longer be observed. The infarcted fraction in a segment with 0.55-relative level can range from 0.70 to 1.00, but there is no possibility to assess this fraction. Note that the breathing also induced an artifactual hypo-perfusion in the normal ventricle model (Figure 5 bottom row).

**Figure 6:**
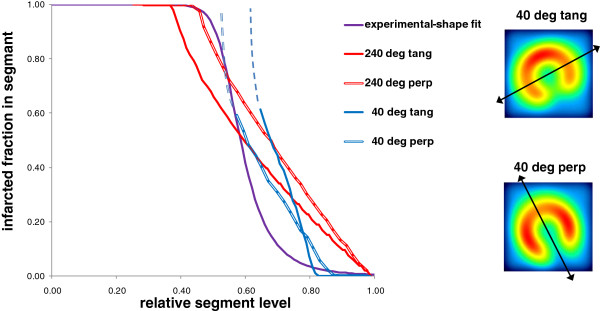
**Impact of the breathing direction on the relative segment level.** Relation between the infarcted fraction in a segment and its relative level derived from the ventricle numerical model for 40° and 240° defect polar extension with a breathing direction (double end arrows) perpendicular or tangential to the defect.

**Figure 7:**
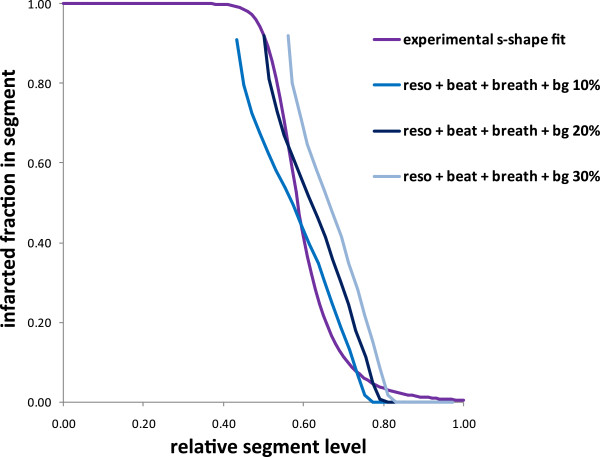
**Impact of the surrounding background on the relative segment level.** Relation between the infarcted fraction in a segment and its relative level derived from the ventricle numerical model for a surrounding background specific activity 10%, 20% and 30% of the normal wall region.

Figure 6 shows the infarcted fraction in a segment as a function of its relative level for a defect polar extension of 40° and 240° and for a breathing motion perpendicular or tangential to the defect. When the defect polar extension decreases below 60°, the segment always intercepts the normal wall. For 40° polar extension of the defect, the infarcted fraction of the segment level of 0.7 is indeterminate from 0.4 to 1. Figure 7 shows the impact of the background level on the curve of the infarcted fraction in a segment as a function of its relative level for the 60° polar extension defect.

### Experimental infarcted fraction in a segment as a function of its measured relative level

The optimal s-curve parameters found in order to have the best correlation between AIF and TTC were *U*_c_ = 0.55 and *n* = 9. This experimental curve was very close to the theoretical predictions derived from the numerical ventricle model (Figures 6 and 7).

### Correlation between AIF and total infarcted fraction observed in the numerical model

**Figure 8:**
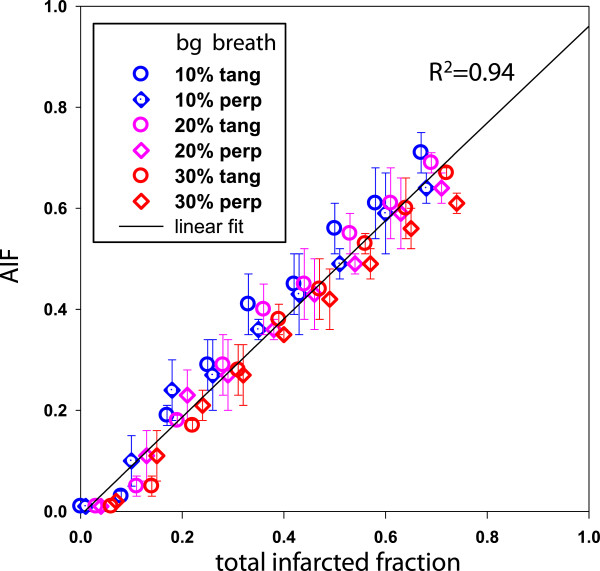
**Correlation between AIF and the total infarcted fraction for various infarction geometries.** Correlation between AIF and the total infarcted fraction derived from the numerical ventricle model for different surrounding background levels (bg) and for the breathing direction tangential or perpendicular to the defect Equations 4 and 5 were used with the experimental parameters *U*_c_ = 0.55 and *n* = 9. The extremities of the error bars correspond to the minimal and maximal AIF obtained by varying the polar position of the defect versus the polar map segmentation. For graphical clarity, all points were simulated for different defect polar extensions.

Figure 8 shows the correlation between AIF and the total infarcted fraction for different surrounding background levels and for different defect polar positions versus the polar map segmentation and versus the breathing directions. AIF was obtained from the numerical ventricle model using Equations 4 and 5 with the experimental parameters *U*_c_ = 0.55 and *n* = 9. The linear regression on all the simulated infarctions was *y* = 0.97x – 0.01; *R*^2^ = 0.94.

### Comparison between SPECT and TTC estimates of the infarct size

**Figure 9:**
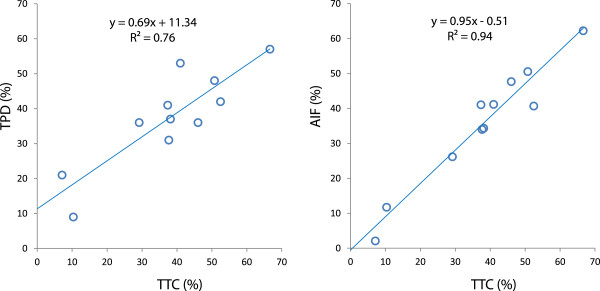
**Correlation of TPD and AIF with TTC staining.** Note that the AIF-TTC linear fit passes through the axis origin.

**Figure 10:**
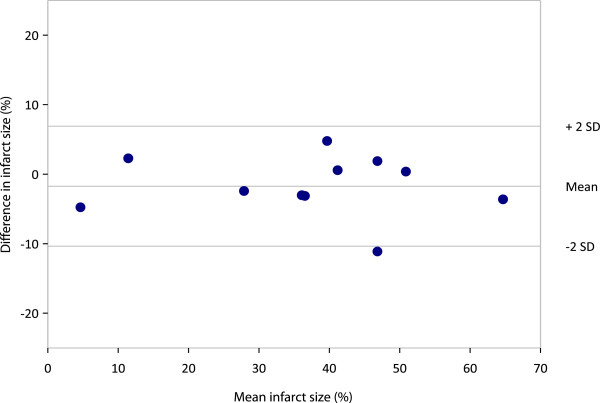
Bland-Altman plot depicting mean difference and limits of agreement (mean ± 2SD) between AIF and TTC.

All the normal mice used to build the normal limits in QGS got 0 for their TPD assessment, while their AIF value was 4.4% ± 1.6%. TTC ranged from 2% to 66%. Mean ± std infarcted fractions were 37.4% ± 13.8%, 37.9% ± 17.5% and 35.6% ± 17.2% for TPD, AIF and TTC, respectively. As shown in Figure 9, AIF provided an excellent correlation with TTC (*R*^2^ = 0.94) which is higher than that found with TPD (*R*^2^ = 0.76). Furthermore, the AIF-TTC linear fit passed through the axis origin. There was no significant bias between AIF and TTC, as demonstrated by the Bland-Altman plot (Figure 10).

## Discussion

A good correlation (*R*^2^ = 0.76) was observed between the post-mortem TTC and the TPD developed by Slomka et al. [13]. However, the study shows that the novel proposed method provided a higher AIF-TTC correlation (*R*^2^ = 0.94) with a linear fit passing through the axis origin. In addition, normal database of myocardial perfusion for mice is no longer required. As a result, AIF is independent of imperfections in the heart reorientation. The suppression of normal databases including different strains and ages in the infarct size assessment process should allow a more precise quantification of the infarct size changes with time. This is an important issue in the field of studies evaluating the impact of regenerative therapies such as stem cells in preclinical models of ischemic heart disease.

The novel method requires only the generation of a 20-segment polar map which is commonly proposed in commercial softwares. Afterwards, the dimensions of the segment contours are measured in arbitrary units on the long axis slices (Figure 1). This can be done by pasting the displayed segment contours into imaging softwares such as paint and PowerPoint, or by simply measuring the contours on a paper copy using a ruler. The segments’ relative level is then rescaled by an s-shaped function taking into account all the effects affecting the final spatial resolution and finally weighted with the segment volume before being summed (Equation 5).

The model optimisation was performed only on 11 mice all bearing LCA infarction geometry, which represents some limitation of the validation. However, the fact that the optimal s-shaped curve experimentally found was very close to the theoretical predictions (Figures 5 and 6), and the fact that the correlation between the AIF and the total infarcted fraction was excellent (Figure 8, *R*^2^ = 0.94) for a wide infarction geometries modelling (i.e. various background levels, various defect sizes, various defect positions versus the breathing and versus the polar map segmentation) strongly supports the validity of the method. Note that the major effects, i.e. system resolution, breathing and surrounding background, are independent of the infarction geometry. The curve pivoting induced by the system resolution and breathing corresponds to a reduction in sensitivity, i.e. the infarcted fraction range (0, 1) in a segment is mapped into the reduced segment relative level range (0.5, 0.8). When the resolution is too altered, a loss of information occurs, i.e. segments with different infarcted fraction can end up to the same relative level (dashed line in Figures 5,6,7). The use of sestamibi leads to an overestimation of infarction area since segments with low perfusion are not always totally necrotic. However, this additional effect altering the effective spatial resolution is accounted for by the fine tuning of the theoretical curve to the experimental data.

Other groups have demonstrated the capability to quantify an infarct size in rats [9-11] and in mice [5,7]. In mice, Wu et al. [5] used a pinhole SPECT system with a spatial resolution of 1.1 mm and Tc-99m-sestamibi as perfusion tracer and the validation was performed against autoradiography. Stegger et al. [8] used a small animal-dedicated PET camera with an effective spatial resolution of 0.7 mm and F-18-FDG. More recently, Wollenweber et al. [7] used a clinical pinhole SPECT system with a spatial resolution of 1.9 mm and Tc-99m-sestamibi. Wu et al. [5] found a good correlation between infarct size (IS) measured with pinhole SPECT and autoradiography (*r* = 0.83; *y* = 0.85x + 2.8). However, they observed an underestimation of ISs relative to autoradiography for small infarcts sizes. This underestimation was attributed to partial volume effect and to physiological motion blurring (heart beats and breathing). Wollenweber et al. [7] observed a systematic underestimation of the infarct size by the pinhole SPECT compared to histology for infarct extension ranging from 15% to 45%). Our results displayed a better correlation (*r* = 0.97), similar to that obtained by Stegger et al. [8] using the state-of-the-art, high-resolution PET camera (*r* = 0.98). These better results are probably at least partially explained by the higher spatial resolution of the Linoview SPECT system (0.6 mm) compared to those of the pinhole SPECT system used by Wu et al. (1.1 mm) or Wollenweber et al. (1.9 mm). Moreover, the Bland-Altman analysis revealed no systematic bias in the wide range of values obtained (Figure 10).

In these previously published studies, perfusion defects were quantified by using specially created softwares based on the generation of circumferential profiles [5,10] or none [8,9]. The novel proposed method only requires a polar map which was obtained in the present study using QPS, a widely available software routinely used in daily clinical practice. This software has been used by Constantinesco et al. [6] who obtained with a multiple pinhole SPECT system an accurate quantification of left ventricular parameters (perfusion and volumes) in normal mice. Unfortunately, in this study however, left ventricular perfusion evaluation was performed only in normal mice.

In our study, the mean activity injected was 173 ± 27 MBq of Tc-99m-sestamibi. This activity is at least two times less than that reported in most studies [5-7]. Tc-99m sestamibi is cleared through the hepatobiliary system, and the gallbladder uptake appears as a focus of radioactivity near the heart in the projection images. This often shadows the apex of the heart in the reconstructed images. To avoid such problem, an intraperitoneal injection of 0.1 μg/kg cholecystokinin (CCK) was performed 2 h after injection of Tc-99m-sestamibi. CCK induces gallbladder contraction, dispersing the activity away from the heart into the small intestine [5]. Tc-99m-tetrofosmin or better Tc-99m-N-DBODC5 has a more rapid liver clearance than Tc-99m-sestamibi [21,22]. The use of those tracers instead of Tc-99m-sestamibi should potentially induce less activity in the gallbladder allowing to skip CCK administration. This issue needs to be further studied.

## Conclusions

The novel developed method applied on SPECT data provides an accurate infarct size assessment after LCA ligature in mice without using a normal perfusion database. The high-resolution Linoview SPECT system is a valuable tool for performing non-invasive and repetitive myocardial perfusion and infarct size evaluations during experimental studies in mice.

## Competing interest

The authors declare that they have no competing interests.

## Authors’ contributions

Each author has contributed significantly to the submitted work: VR: design, mouse experiments, data acquisition, analysis, interpretation and co-writing; MD: design, mouse experiments, data acquisition and co-writing; SW: design, analysis, mouse experiments, data acquisition, interpretation and co-writing; RL: analysis, interpretation and co-writing. YB: design and co-writing; FJ and J-LV: design and critical revision of the manuscript. All authors read and approved the final manuscript.

## Authors’ information

MD is a Télévie research assistant, YB is the research director of the Fonds National de la Recherche Scientifique et Médicale (FNRS), Belgium.
